# A Complete Pathway Model for Lipid A Biosynthesis in *Escherichia coli*

**DOI:** 10.1371/journal.pone.0121216

**Published:** 2015-04-28

**Authors:** Akintunde Emiola, John George, Steven S. Andrews

**Affiliations:** 1 School of Health, Sports and Bioscience, University of East London, London, United Kingdom; 2 Fred Hutchinson Cancer Research Center, Seattle, Washington, United States of America; University of Cambridge, UNITED KINGDOM

## Abstract

Lipid A is a highly conserved component of lipopolysaccharide (LPS), itself a major component of the outer membrane of Gram-negative bacteria. Lipid A is essential to cells and elicits a strong immune response from humans and other animals. We developed a quantitative model of the nine enzyme-catalyzed steps of *Escherichia coli* lipid A biosynthesis, drawing parameters from the experimental literature. This model accounts for biosynthesis regulation, which occurs through regulated degradation of the LpxC and WaaA (also called KdtA) enzymes. The LpxC degradation signal appears to arise from the lipid A disaccharide concentration, which we deduced from prior results, model results, and new LpxK overexpression results. The model agrees reasonably well with many experimental findings, including the lipid A production rate, the behaviors of mutants with defective LpxA enzymes, correlations between LpxC half-lives and cell generation times, and the effects of LpxK overexpression on LpxC concentrations. Its predictions also differ from some experimental results, which suggest modifications to the current understanding of the lipid A pathway, such as the possibility that LpxD can replace LpxA and that there may be metabolic channeling between LpxH and LpxB. The model shows that WaaA regulation may serve to regulate the lipid A production rate when the 3-deoxy-D-*manno*-oct-2-ulosonic acid (KDO) concentration is low and/or to control the number of KDO residues that get attached to lipid A. Computation of flux control coefficients showed that LpxC is the rate-limiting enzyme if pathway regulation is ignored, but that LpxK is the rate-limiting enzyme if pathway regulation is present, as it is in real cells. Control also shifts to other enzymes if the pathway substrate concentrations are not in excess. Based on these results, we suggest that LpxK may be a much better drug target than LpxC, which has been pursued most often.

## Introduction

Lipopolysaccharide (LPS) is a glycolipid that forms the major component of the outer leaflet of the outer membrane of most Gram-negative bacteria. It occurs with roughly 1 million copies in *Escherichia coli* cells, covering about 75% of the cell surface area **[1],[2],[3]**. LPS helps stabilize these membranes, protects them from chemical attack, and promotes cell adhesion to various surfaces **[4]**. It elicits a strong immune response in humans and other animals (and is a main contributor to Gram-negative septic shock), getting detected at picomolar levels by the innate immune system’s TLR4 protein **[5]**. These attributes have made the study of LPS important to the fields of immunology, bacteriology, and drug discovery **[1],[2],[5],[6],[7],[8]**.

**Fig 1:**
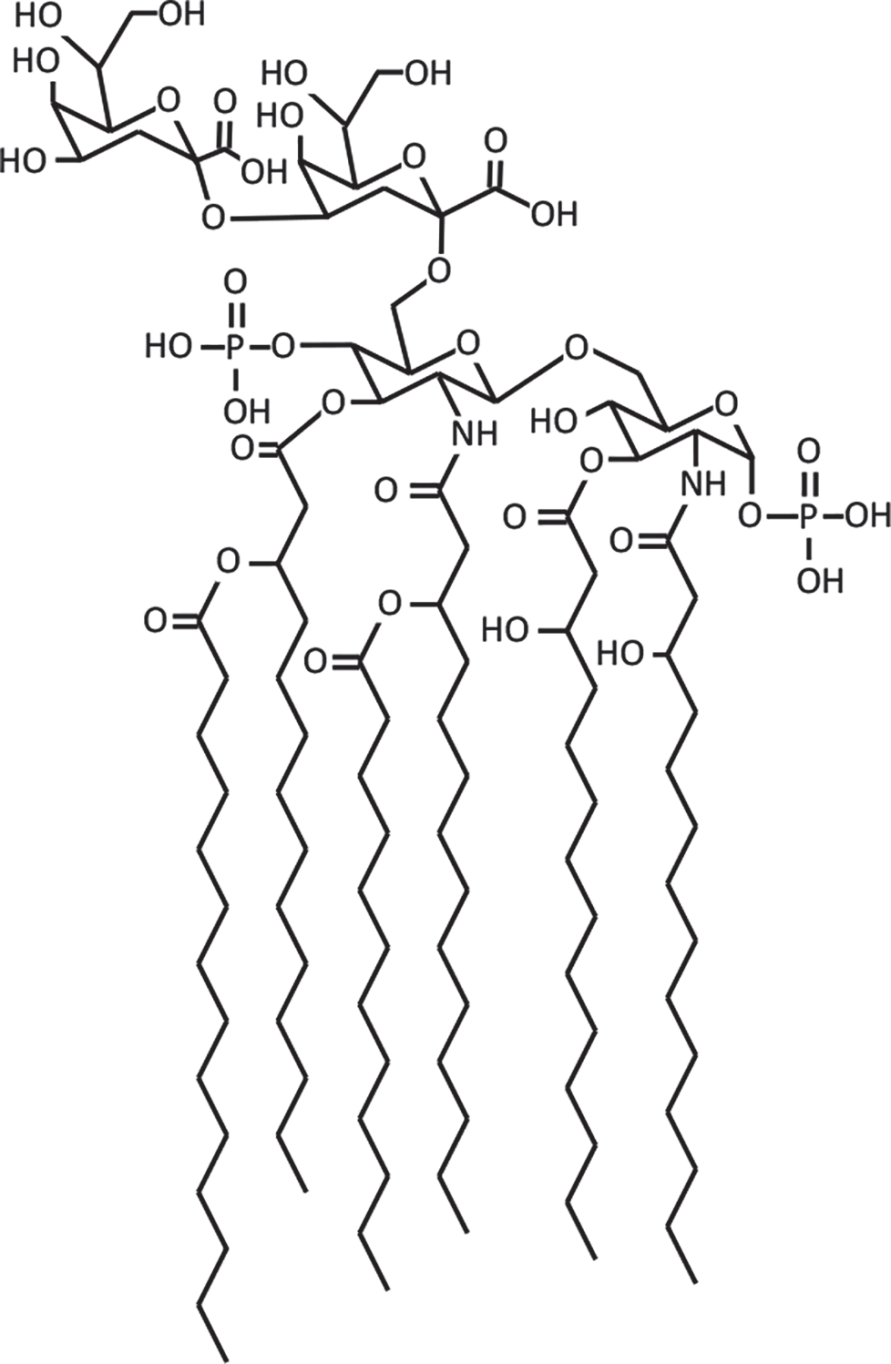
Structure of KDO_2_-lipid A. The top two sugars are KDO groups, which are part of the core oligosaccharide, while the remainder of the structure represents lipid A.

LPS comprises three components: lipid A, core oligosaccharide, and O-antigen **[1],[5]**. The lipid A, or endotoxin component (Fig 1) includes six hydrophobic acyl chains that reside in the outer leaflet of the bacterial outer membrane. These are connected together by a glucosamine and phosphate head group. In most Gram-negative bacteria, including *E*. *coli*, this head group connects to a pair of KDO sugar residues (3-deoxy-D-*manno*-oct-2-ulosonic acid) **[1]**. These KDO residues connect to several additional sugar residues, and sometimes also to phosphate, pyrophosphorylethanolamine, or phosphorylcholine residues, which together form the core oligosaccharide **[5]**. This core then connects to the O-antigen, which is a long polysaccharide that varies widely between different bacterial species and different strains within each species **[5]**. Of these three components, the lipid A moiety is of particular interest because it is the only component that is essential for cell viability and is highly conserved **[5]**. These also make its biosynthetic pathway an attractive target for new antibiotics **[5],[9],[10],[11]**.

**Fig 2:**
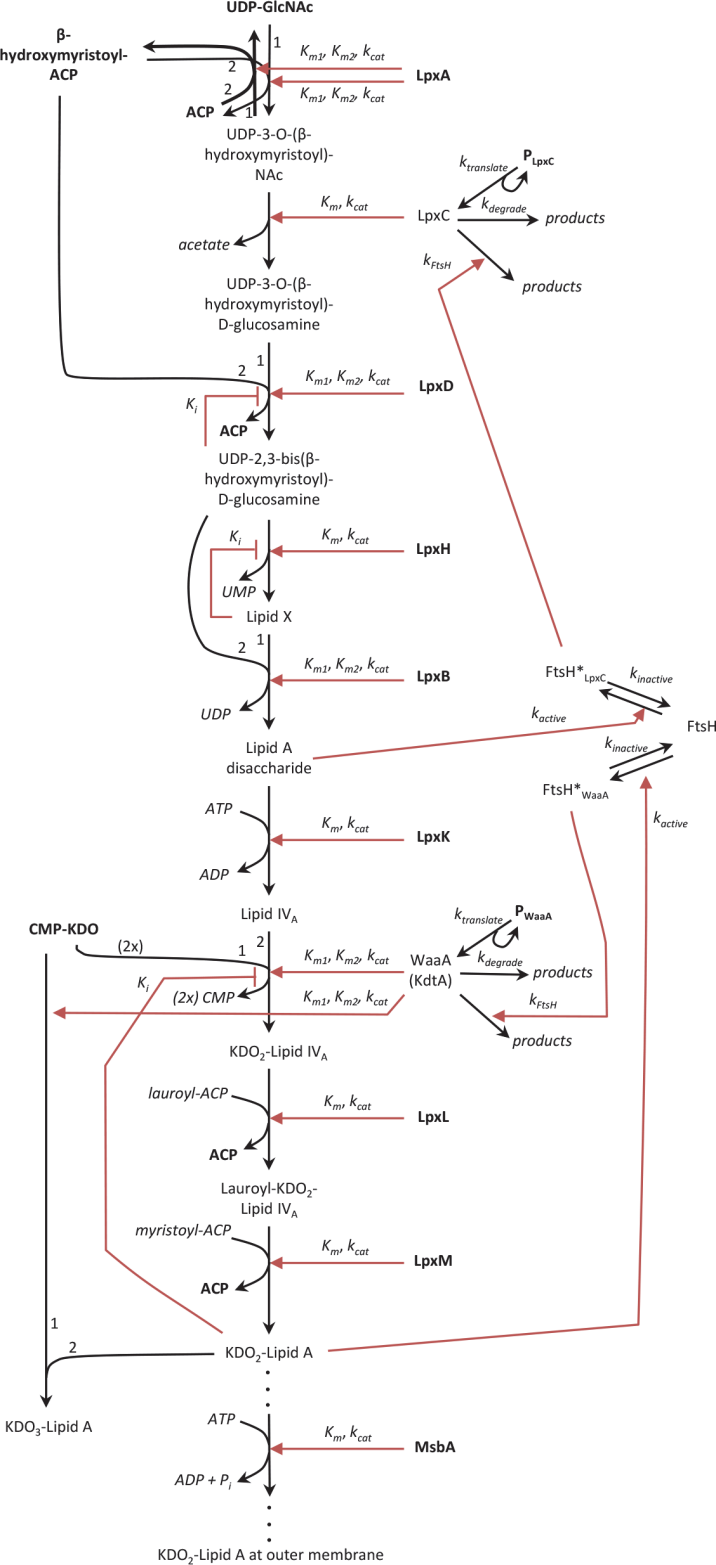
Model of the *E*. *coli* KDO_2_-lipid A biosynthesis pathway. Enzymes and metabolites are shown with three text styles: upright bold indicates that these concentrations are fixed, upright plain indicates that these concentrations vary, and italics indicates that these species are not included in the model explicitly. Black arrows with barbed heads represent chemical reactions in which reactants are converted to products. Red arrows with closed heads represent enzymatic influences on chemical reaction rates, and red arrows with T-bar heads represent inhibitory influences. Variables represent model parameters. Numbers next to black arrows for bi-substrate reactions show which substrate is designated number 1 and number 2.

The lipid A biosynthesis pathway architecture, shown in Fig 2 and described in more detail below, has been investigated thoroughly through several decades of careful experimentation **[1],[2],[5],[7],[8]**. However, it has received remarkably little quantitative analysis, which is essential for testing the internal consistency of models and for investigating pathway regulation mechanisms. In one modeling study, Kenanov et al. **[12]** investigated the elementary flux modes (unbranched paths through the metabolic chemical reaction network, not including regulatory interactions) for the biosynthesis of all *E*. *coli* lipids. They found close agreement between the predicted and experimental viability of knock-out mutants. This supported the lipid biosynthesis pathway architecture that they used, which is also the one that is commonly accepted and that which we assume in this work. In other modeling work, we recently simulated the chemical kinetics of the first two steps of lipid A biosynthesis **[13]**. We found that the second enzyme (LpxC) has sufficient catalytic activity to overcome the first enzyme’s unfavourable equilibrium constant. We are not aware of any other computational models of lipid A biosynthesis.

Here, we build on the prior experimental and modeling work to present a quantitative model of the central steps of lipid A biosynthesis. This model is specific to *E*. *coli* because *E*. *coli* has been the subject of most lipid A research. However, the lipid A biosynthesis pathway is well conserved across Gram-negative bacteria **[5]**, so our model may be applicable to other Gram-negative bacteria as well. We used published parameters where possible and estimated others as required. Our model can reproduce the observed lipid A production rate and agrees reasonably well with results from several lipid A biosynthesis experiments. These include data that correlated LpxC half lives and cell generation times **[14],** and our own experiments on LpxK overexpression. Our model also disagrees with several experimental results. These disagreements highlight potentially interesting biological behaviour such as metabolic channeling and additional sources of pathway regulation.

## Methods

### Simulations

Simulations were performed with non-spatial deterministic methods using the COPASI software **[15]**. This level of detail was determined to be adequate because preliminary simulations using spatial stochastic simulations (with Smoldyn **[16]**) and non-spatial stochastic simulations (with StochKit **[17]**) yielded essentially identical results. The COPASI files are available as supplementary information and will be submitted to the BioModels database.

### Experimental procedures

#### Bacterial strain and growth conditions

An *E*. *coli* K-12 strain AG1 (*recA1*, *endA1*, *gyrA96*, *thi-1*, *hsdR17(r*_*K*_^*-*^ *m*_*K*_^*+*^ *)*, *supE44*, *relA1*) containing a plasmid (pCA24N) **[18]** bearing *E*. *coli* LpxK-GFP gene fusion (to the C-terminus) was obtained from the National BioResource Project (NIG) Japan. Cells were grown at 30°C in LB media (10g tryptone, 5g yeast extract, 5g NaCl per liter) containing 20 μg/ml of chloramphenicol and when required, protein expression was induced using IPTG (Sigma, UK).

#### Preparation of cell extracts

Cell extracts were prepared as described previously **[19],[20]**. Briefly, an overnight culture was inoculated into fresh LB containing different concentrations of IPTG at an OD_600_ of 0.05 and grown to mid log phase (OD_600_ = 0.5). The respective cultures were normalized to the same OD_600_ of 0.5. 3 ml of normalized culture was centrifuged at 13,000 rpm for 1 min and the cell pellets re-suspended in 100 μl of 2x Laemmli sample buffer (Sigma, UK). The samples were heated for 10 min prior to centrifugation for 5 min. The supernatants were collected for Western blot analysis.

#### Western blot

20 μl of each sample were loaded onto a 10% SDS-polyacrylamide gel. Following electrophoresis, proteins were transferred to a PVDF membrane using the Bio-Rad Trans-Blot Turbo system. An LpxC antiserum generated in rabbit (a generous gift from Prof. Franz Narberhaus) and a secondary anti-rabbit peroxidase-linked antibody (Sigma, UK) were used for immunodetection at dilutions of 1:20000 and 1:10000 respectively. Blots were developed using the ECL chemiluminiscent reagents (Bio-Rad) and the signals detected using the ChemiDoc MP system (Bio-Rad).

### Model architecture

#### Lipid A biosynthesis pathway

*E*. *coli* lipid A biosynthesis proceeds through nine enzyme-catalyzed steps, which are sometimes referred to as the Raetz pathway **[2],[7]** (Fig 2). All of these enzymes are constitutively expressed **[5]**. The pathway has been reviewed several times recently **[1]**,**[2],[5] [7],[8]**, so we only provide a brief summary here, while focusing on the features that are particularly salient to our model.

Lipid A biosynthesis begins with the UDP-N-acetylglucosamine (UDP-GlcNAc) and β-hydroxymyristoyl-ACP substrates. Both substrates are consumed in other metabolic pathways as well **[21]**: UDP-GlcNAc is a substrate in peptidoglycan synthesis **[22],[23]** and β-hydroxymyristoyl-ACP is a precursor for phospholipid metabolism **[24],[25],[26]**.

The first three steps of the lipid A pathway occur in the cytoplasm. First, LpxA (EC 2.3.1.129) acylates UDP-GlcNAc with β-hydroxymyristoyl-ACP. This reaction has an unfavourable equilibrium constant of 0.01 *in vitro* **[27]**, suggesting that the reaction products are not committed to proceed on through the lipid A pathway but may instead revert back into the pathway substrates. The product is then deacetylated by LpxC (EC 3.5.1.108) in an essentially irreversible reaction, making this the first committed pathway step **[19],[28],[29],[30]**. For this and other reasons, LpxC is likely to be a primary biosynthesis control point **[27]** (and is a prime drug target), as discussed below. The third pathway enzyme, LpxD (EC 2.3.1.191), incorporates a second hydroxymyristate moiety onto the lipid A precursor **[31],[32]**. LpxD is similar to LpxA in that they are acyltransferases, and consume the same β-hydroxymyristoyl-ACP substrate **[32]**. Both LpxD reaction products inhibit the LpxD reaction, acting as either competitive or non-competitive inhibitors against each substrate **[32]**. We simplified this by only including non-competitive inhibition by UDP-2,3-bis(β-hydroxymyristoyl)-D-glucosamine. Ignoring inhibition by ACP had minimal effect on our results because we fixed its concentration.

The fourth and fifth lipid A biosynthesis steps are catalyzed by the peripheral membrane proteins LpxH (EC 3.6.1.54) **[33]** and LpxB (EC 2.4.1.182) **[34]**. LpxH cleaves most of the UDP moiety to leave just a single phosphate on the remaining lipid portion, which is called lipid X. Feedback regulation has not been proposed before for LpxH but proved necessary for our model to achieve steady-state behaviour, as described below. Then, LpxB combines lipid X with the preceding lipid metabolite, UDP-2,3-bis(β-hydroxymyristoyl)-D-glucosamine, to form lipid A disaccharide **[34]**.

The remaining four steps of lipid A biosynthesis are catalyzed by integral membrane enzymes. LpxK (EC 2.7.1.130) is a kinase that phosphorylates lipid A disaccharide to produce lipid IV_A_ **[35],[36]**. Remarkably, Lipid IV_A_ has been reported to be an endotoxin agonist in mouse cells and an endotoxin *antagonist* in human cells **[37]**. Next, WaaA (previously called KdtA, EC 2.4.99.12/13) sequentially transfers two KDO sugar residues to lipid IV_A_ to produce KDO_2_-lipid IV_A_ **[38],[39],[40]**. WaaA has low substrate specificity, with the result that KDO_2_-lipid A can act as a competitive inhibitor for this reaction **[40]**, or as another possible WaaA substrate **[39],[40]**; in the latter case, the reaction produces “alternate lipid A”, which we define as having more than 2 KDO sugar residues. In addition, the WaaA reaction has been shown to be reversible, based on the finding that *in vitro* combinations of the reaction products (enzyme, cytidine 5'-monophosphate (CMP), and KDO_2_-lipid IV_A_) led to detectible concentrations of the KDO-lipid IV_A_ intermediate **[40]**. However, the authors only observed trace quantities of lipid IV_A_ even after prolonged incubations, thus, the forward reaction is likely to be strongly thermodynamically favourable, and in which case, the *in vivo* back-reaction rate is probably negligible. For this reason, our model treats WaaA catalysis as being irreversible. Finally, the “late acyltransferases,” LpxL (EC 2.3.1.-) **[41]** and LpxM (EC 2.3.1.-) **[42],[43]**, incorporate lauroyl and myristoyl chains to the KDO_2_-lipid IV_A_, thus giving the final KDO_2_-lipid A product six acyl chains. Cells are still viable without LpxM, or without LpxL and with overexpressed LpxM **[44]**. In cold-adapted *E*. *coli*, the LpxL function is replaced by LpxP (EC 2.3.1.-), which incorporates a palmitoleate instead of the laurate, presumably as a way of adjusting membrane fluidity **[45]**.

After synthesis, KDO_2_-lipid A is joined to core oligosaccharide and then flipped from the inner leaflet of the inner membrane to the outer leaflet of the inner membrane by MsbA (EC 3.6.3.39), an ABC transporter **[2],[46]**. Next, several enzymes add the O-antigen to form LPS, and then transport the LPS on to the outer leaflet of the outer membrane **[7]**.

#### Lipid A biosynthesis regulation

Lipid A synthesis is regulated, at least in part, through controlled degradation of LpxC **[47],[48],[49]** and WaaA **[50]**, both performed by FtsH (EC 3.4.24.—). FtsH is an integral membrane AAA-type metalloprotease that degrades a wide variety of proteins. These include heat shock transcription factor RpoH (σ^32^), phage λ proteins CII and CIII, and many misfolded proteins **[51],[52]**. FtsH is an essential protein due to its role in regulating LpxC **[47],[53]**. Lipid A biosynthesis regulation is less well established than is the synthetic pathway, so we describe these aspects of our model in more detail.

We assume that FtsH can reversibly convert between an inactive state, an active state for degrading LpxC, and a different active state for degrading WaaA (denoted FtsH, FtsH*_LpxC_, and FtsH*_WaaA_, respectively). This assumption of substrate-specific FtsH activation is supported by several findings: (*i*) FtsH degradation of RpoH and λ CII is substrate-specific, accomplished through separate adapter proteins **[52],[54]**, (*ii*) neither increased nor decreased FtsH degradation of LpxC have a significant effect on the intracellular concentration of RpoH **[47]** or the activity of WaaA **[19]**, and (*iii*) LpxC is degraded more slowly at higher temperatures whereas WaaA is degraded more rapidly at higher temperatures **[14],[50]**. Substrate-specific activation of FtsH for LpxC may occur through YciM acting as an LpxC adapter protein **[55]**.

**Fig 3:**
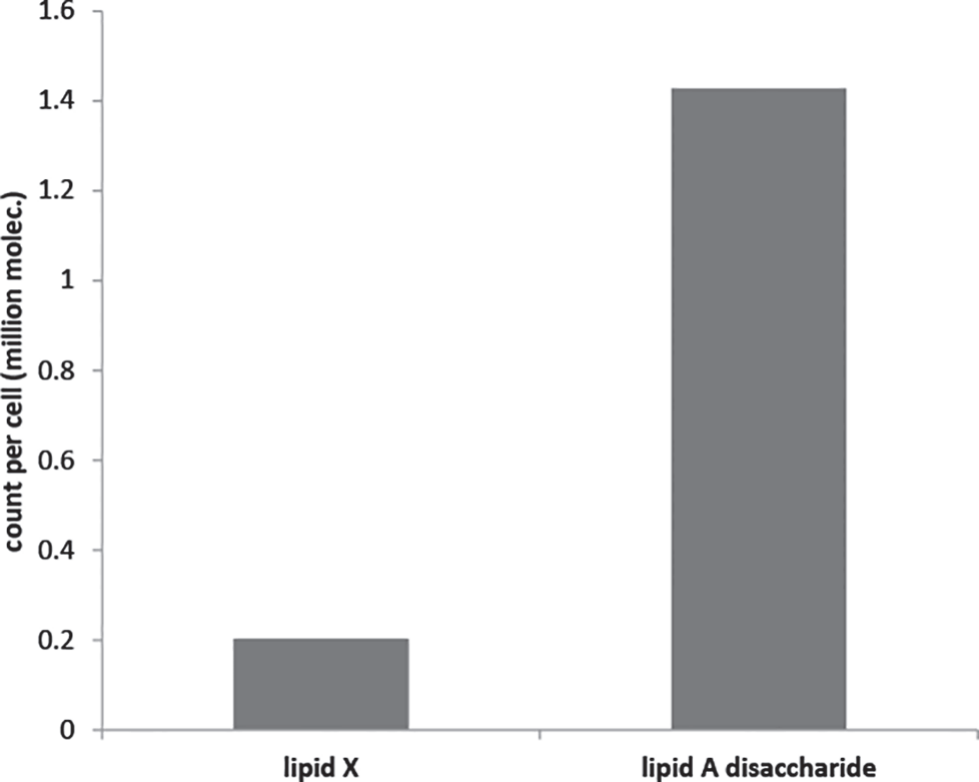
Lipid A disaccharide accumulation. Bars indicate intracellular counts of lipid X and lipid A disaccharide at the end of a single cell generation from preliminary model results. The model used the scheme shown in Fig 2 and parameters listed in Table 1, with the exceptions: the FtsH count was set to zero, the LpxC and WaaA counts were set to their steady-state levels in the absence of FtsH degradation (1540 and 978, respectively), and all metabolites were started with zero molecules. Although not shown here, the lipid X count had stabilised at a constant level, while the lipid A disaccharide count was increasing at a constant rate of 924 molecules/s.

We assume that the regulatory signal that directs FtsH degradation of LpxC arises from the concentration of lipid A disaccharide. Again, this is based on several findings. First, strains that have decreased LpxA, LpxC, or LpxD function, whether through temperature-sensitive mutants or chemical inhibition, exhibit decreased lipid A content and slowed LpxC degradation **[11],[19],[27],[56]**. These suggest that the feedback source is downstream of LpxD. Second, chemically inhibiting CMP-KDO production **[57]** blocks the lipid A biosynthesis pathway at the WaaA point. This was found to cause lipid IV_A_ accumulation but did not affect LpxC activity **[19]**. This suggests that the feedback source is upstream of lipid IV_A_. Three metabolites fit these two criteria, UDP-2,3-bis(β-hydroxymyristoyl)-D-glucosamine, lipid X, and lipid A disaccharide. Of these, UDP-2,3-bis(β-hydroxymyristoyl)-D-glucosamine was already regulated by product inhibition, which makes its concentration a poor indicator of pathway flux and hence a poor candidate. Preliminary simulations that represented FtsH knock-out mutants, and hence did not include FtsH regulation, exhibited lipid A disaccharide accumulation (Fig 3). This suggested that the feedback source is lipid A disaccharide. Guided by these arguments, we experimentally investigated the effect of over-expressing LpxK; those results further supported the lipid A disaccharide choice, as explained below.

Finally, we assume that the regulatory signal for FtsH degradation of WaaA arises from KDO_2_-lipid A that is inside the inner membrane. We chose KDO_2_-lipid A rather than its precursors because the two enzymes downstream of WaaA, which are LpxL and LpxM, are non-essential **[44]**, making their substrates unlikely activators. Also, we chose KDO_2_-lipid A before it has been transported to the outer membrane, rather than afterwards, because bacterial cells are known to regulate excess lipid A in their outer membrane by shedding it into the environment **[58]**.

Other regulatory signals impinge upon the lipid A biosynthesis pathway as well but are outside of the scope of this work. In particular, modifications to the FabZ and FabI enzymes, used for phospholipid synthesis, have been shown to affect the concentration of LpxC and hence affect the rate of lipid A production **[20],[24],[47]**. By assuming that FabZ and FabI are maintained at wild-type conditions, and that there are no feedback processes from the lipid A pathway that affect the FabZ or FabI regions of the phospholipid pathway, we were able to legitimately ignore these additional regulatory signals in this work.

### Model Equations and Parameters

We modeled the interactions of individual substrates and enzymes for a single *E*. *coli* cell at steady state. We assumed that the volume of a cell is 6.7×10^–16^ liters **[59]** (thus 1 molecule represents 2.5 nM, 1000 molecules represents 2.5 μM, and 10^6^ molecules represents 2.5 mM). Unless specified otherwise, we assumed a 30 minute (1800 s) doubling time, which is the experimental value in rich media **[60], [61]**. For the most part, we did not account for protein synthesis, protein degradation, or cell volume growth during a cell generation. These approximations are legitimate because metabolic enzyme concentrations for constitutive enzymes likely remain constant over the course of the cell cycle. Table 1 lists our model parameters, along with the relevant data sources.

**Table 1:** Abundance and kinetic parameters of lipid A biosynthesis model.

Species	Location	Abundance(molec./cell)	*K*_*m*_ or *K*_*m*1_ (mM)	*K*_*m*2_(mM)	*k*_*cat*_(s^-1^)	Notes and other parameters
UDP-GlcNAc		2,000,000				excess concentration
β-hydroxymyristoyl-ACP		2,000,000				excess concentration
CMP-KDO		2,000,000				excess concentration
ACP		1024^a^				actual concentration
LpxA	cyto.	664^a^	0.82^f^	0.0016^m^	7.17^f^	
			0.82	0.0016	717	back reaction
LpxC	cyto.	385^b^	0.00019 ^g^		3.3^l^	*k*_*translate*_ = 0.148 s^-1^ *k*_*degrade*_ = 9.62×10^–5^ s^-1^
LpxD	cyto.	453^a^	0.0025^h^	0.0032^h^	23^h^	*K*_*i*_ = 0.0094 mM^h^
LpxH	p.m.	177^c^	0.0617^c^		47	*K*_*i*_ = 0.015 mM
LpxB	p.m.	384^d^	0.287^d^	0.381^d^	129	
LpxK	i.m.	432	0.04^i^		2.1	
WaaA	i.m.	153^a^	0.088^j^	0.052^j^	16.7	*K*_*i*_ = 0.0317 mM *k*_*translate*_ = 0.176 s^-1^ *k*_*degrade*_ = 1.8×10^–4^ s^-1^
			0.088^j^	0.052^j^	1.9	substrate is KDO_2_-lipid A
LpxL	i.m.	928^e^	0.015^k^		131^k^	
LpxM	i.m.	3720	0.00275		0.6	
MsbA	i.m.	206^a^	0.021		166	
FtsH	i.m.	579^a^				
FtsH*_LpxC_	i.m.	-	*k*_*FtsH*_ = 2.0 mM^-1^s^-1^			*k*_*active*_ = 0.14 mM^-1^s^-1^ *k*_*inactive*_ = 0.1 s^-1^
FtsH*_WaaA_	i.m.	-	*k*_*FtsH*_ = 6.8 mM^-1^s^-1^			*k*_*active*_ = 32.3 mM^-1^s^-1^ *k*_*inactive*_ = 0.1 s^-1^

These values are for *E*. *coli* cells in rich media. Location abbreviations are: cyto. for cytoplasm, p.m. for peripheral membrane, and i.m. for integral membrane (locations are not part of the model). Data estimation methods are presented in the main text. Data are from: (a) **[62]**, (b) **[13]**, (c) **[33]**, (d) **[63]**, (e) **[41]**, (f) **[76]**, (g) **[94]**, (h) **[32]**, (i) Fig 5 in **[35]**, (j) **[40]**, (k) **[95]**, (l) **[96]**, (m) **[27]**. Parameters that do not have citations are discussed in the main text.

#### Substrate concentrations

We investigated lipid A metabolism with either excess or limiting concentrations of the UDP-GlcNAc, β-hydroxymyristoyl-ACP, and CMP-KDO substrates. The stoichiometric ratios of these substrates to lipid A are 2:1, 4:1, and 2:1, respectively, due to consumption of multiple copies and/or lipid dimerization by LpxB. The first two of these substrates are also precursors for other biosynthetic pathways **[21], [22], [23], [25]**, so their relative concentrations are controlled by factors outside our simulation. We kept their levels constant at 2 million molecules (5 mM) throughout most simulations, which led to substrate saturation conditions. Additionally, we fixed the ACP level to 1024 molecules, based on proteomic results **[62]**.

#### Enzyme abundance

We used LpxA, LpxD, WaaA, MsbA, and FtsH protein copy numbers from mass spectrometry proteomic data collected on *E*. *coli* cytosolic fractions **[62]**. Several of these are integral membrane proteins, so their experimental protein counts are likely to be lower limits for their true counts in a cell. We used the same proteomic data source for the copy number of LpxM **[62]**, another integral membrane protein, but then increased the count 20-fold as described below. We calculated LpxC, LpxH, and LpxB protein counts from protein purification data **[13][33][63]**, along with the assumption that an average *E*. *coli* cytoplasm contains approximately 1.9 million protein molecules **[64]**. Similarly, we calculated LpxL protein counts from protein purification experiments **[41]** and the estimate that an *E*. *coli* membrane contains 580,000 proteins **[64]**.

We estimated the LpxK protein count using information about MsbA. These proteins are co-transcribed **[65]**, implying that their transcripts are synthesized at similar rates. Thus, differences in their expression rates depend on the relative stability of their transcripts and on the translation rates for individual proteins. The MsbA and LpxK transcript half-lives have been reported as 3.2 min and 3.8 min respectively **[66]**, from which we calculated their mean lifetimes as 277 s and 329 s. Given that the average translation rate is 20 amino acids per second **[67]**, and that they comprise 582 **[68]** and 328 amino acids respectively **[36]**, it should take about 29 s and 16 s for their translations. This means that about 9.6 MsbA proteins and 20 LpxK proteins are translated over the lifetimes of their respective mRNAs. Thus, LpxK is likely to be synthesized about 2.1 times faster than MsbA. Both LpxK and MsbA are membrane proteins, so we assumed that they had similar degradation rates **[69]**. This meant that the synthesis rate ratio also represented the protein concentration ratio. MsbA has an abundance of about 206 molecules per *E*. *coli* cell **[62]**, from which we calculated that the LpxK abundance is about 432 molecules.

#### Enzyme kinetics

We modeled all pathway reactions using single-substrate or bi-substrate Michaelis-Menten mechanisms. We ignored reaction reversibility in most cases. This can lead to misleading results in metabolic models because it ignores feedback effects that arise from product inhibition and hence can prevent models from attaining a steady-state **[70],[71]**. However, it was legitimate here because our model includes regulatory feedbacks that extend over most of the pathway length. These are alternative ways to enable models attain steady-state, and in fact are typically more effective than reversible reactions **[72]**. Also, most of the lipid A reactions are likely to be nearly irreversible, due to either favourable energetics or much more abundant substrates than products (e.g. the phosphorylation reaction catalyzed by LpxK is effectively irreversible because ATP is abundant in cells whereas ADP is not).

We used single-substrate Michaelis-Menten kinetics for the LpxC, LpxK, LpxL, LpxM, and MsbA steps. Here, the metabolite flux is

$$\frac{d\left[\text{P}\right]}{dt}=-\frac{d\left[\text{S}\right]}{dt}=\frac{k_{cat}\left[\text{E}\right]\left[\text{S}\right]}{K_{m}+\left[\text{S}\right]}$$ (1)

where [S] is the substrate concentration, [P] is the product concentration, [E] is the total enzyme concentration, *k*_*cat*_ is the enzyme catalytic rate constant, and *K*_*m*_ is the Michaelis constant. Most of these *k*_*cat*_ and *K*_*m*_ values have been published using data from *in vitro* experiments (Table 1), although we needed to estimate a few of them. (*i*) The specific activity of LpxK in crude *E*. *coli* membrane extract was estimated to be 22 nmol/min/mg in a plasmid-containing strain but 7-fold lower in wild type **[65]**. There are about 432 LpxK molecules per cell and *E*. *coli* membranes include about 580,000 individual proteins **[64]**, so the LpxK purity in crude membrane is about 0.074%. Thus, the pure wild-type specific activity is about 4 μmol/min/mg, from which *k*_*cat*_ is about 2.1 s^-1^. We estimated that the LpxK *K*_*m*_ value is 40 μM from a figure presented by Ray and Raetz **[35]**. Although *K*_*m*_ estimations from crude samples are prone to inaccuracies when the substrate can be catalyzed by other enzymes in the lysates, there is no evidence of such competition for the LpxK substrate. (*ii*) We estimated the LpxM catalytic rate constant, *k*_*cat*_, as 0.6 s^-1^ from the specific activity of the enzyme in crude lysates **[42]**, much as we did for LpxK. We estimated the LpxM *K*_*m*_ value from data shown in Fig 6 of Clementz *et al*. **[43]**. To do so, we simulated Clementz et al.’s experiment using COPASI **[15]**, with the same enzyme and substrate concentrations that they used (0.1 μg/mL and 25 μM for protein and KDO_2_-lipid IV_A_ respectively in a 20 μL reaction mixture), from which we identified the *K*_*m*_ value that corresponded to their figure results at a time of 30 minutes. (*iii*) We treated the MsbA catalyzed translocation of KDO_2_-lipid A across the inner membrane as another Michaelis-Menten process, setting its *K*_*m*_ value to 0.021 mM and its *k*_*cat*_ value to 166 s^-1^, based on data shown in Fig 6 of **[73]**.

We used single-substrate Michaelis-Menten kinetics with inhibition for the LpxH enzyme. In this case, the metabolite flux is

$$\frac{d\left[\text{P}\right]}{dt}=-\frac{d\left[\text{S}\right]}{dt}=\frac{k_{cat}\left[\text{E}\right]\left[\text{S}\right]}{\left(K_{m}+\left[\text{S}\right]\right)\left(1+\frac{\left[\text{P}\right]}{K_{i}}\right)}$$ (2)

where *K*_*i*_ is the inhibition constant and the other parameters are the same as in Eq 1. Assays conducted on LpxH purified to 60% homogeneity displayed a specific activity of 63.2 μmol/min/mg **[33]**. This implies that the pure enzyme specific activity is about 105 μmol/min/mg, which is combined with the LpxH molecular weight of 26.8 kDa **[33]**, to yield its *k*_*cat*_ as about 47 s^-1^. The estimation of our *K*_*i*_ value is discussed below.

We used bi-substrate Michaelis-Menten kinetics for the forward LpxA, reverse LpxA, LpxB, and WaaA catalysis of KDO_2_-lipid A steps. Treating the forward and reverse LpxA reactions as independent irreversible reactions is legitimate in non-spatial models, such as ours, because doing so does not introduce any new approximations (although, this is not true for spatial models **[74]**). Most bi-substrate enzymatic reactions follow either a sequential or ping-pong mechanism **[75]**. In the sequential mechanism, the enzyme forms a ternary complex with both substrates before catalyzing the reaction. In the ping-pong mechanism, the enzyme binds one substrate, forms one product, and then binds the second substrate and forms the second product. The only bi-substrate reactions in the lipid A pathway that have been investigated in sufficient detail to determine mechanisms are the steps catalyzed by LpxA **[76]** and LpxD **[32]**, both of which were found to follow the sequential mechanism. Lacking further experimental evidence, we assumed sequential mechanisms for the other bi-substrate reactions in the lipid A pathway as well. The sequential mechanism metabolite flux is

$$\frac{d\left[\text{P}\right]}{dt}=-\frac{d\left[{\text{S}}_{1}\right]}{dt}=-\frac{d\left[{\text{S}}_{2}\right]}{dt}=\frac{k_{cat}\left[\text{E}\right]\left[{\text{S}}_{1}\right]\left[{\text{S}}_{2}\right]}{\left(K_{m_{1}}+\left[{\text{S}}_{1}\right]\right)\left(K_{m_{2}}+\left[{\text{S}}_{2}\right]\right)}$$ (3)

where [S_1_] and [S_2_] are the two substrate concentrations, with respective Michaelis-Menten constants *K*_*m*1_ and *K*_*m*2_. We needed to estimate these parameters in some cases. (*i*) The LpxA kinetic parameters were determined previously for the forward reaction **[27], [76]**, but not for the reverse reaction. Thus, we assumed the same *K*_*m*_ values for the reverse reaction as for the forward reaction (we set *K*_*m*_ of ACP to that for β-hydroxymyristoyl-ACP, and *K*_*m*_ of UDP-3-O-[β-hydroxymyristoyl]-NAc to that for UDP-GlcNAc), based upon the likelihood that the enzyme binding affinities are not substantially affected by the acyl group transfer. However, we set the reverse reaction *k*_*cat*_ value to 100 times that of the forward reaction to account for the reaction’s unfavourable equilibrium constant of approximately 0.01 **[27]**. (*ii*) We estimated the LpxB *k*_*cat*_ value by starting from the specific activity of LpxB purified to near homogeneity **[63]**, much as we did for LpxH, which resulted in a *k*_*cat*_ value of 129 s^-1^. (*iii*) The WaaA specificity for KDO_2_-lipid A is 8.7 fold lower than for lipid IV_A_ **[40]**. To account for this, we reduced the *k*_*cat*_ value for the former reaction by 8.7 fold while keeping other reaction constants the same.

Finally, we used bi-substrate Michaelis-Menten kinetics with inhibition for the LpxD and WaaA steps. Assuming the sequential mechanism again, which was shown to be the correct mechanism for LpxD, the metabolite flux is

$$\frac{d\left[\text{P}\right]}{dt}=-\frac{d\left[{\text{S}}_{1}\right]}{dt}=-\frac{d\left[{\text{S}}_{2}\right]}{dt}=\frac{k_{cat}\left[\text{E}\right]\left[{\text{S}}_{1}\right]\left[{\text{S}}_{2}\right]}{\left(K_{m_{1}}+\left[{\text{S}}_{1}\right]\right)\left(K_{m_{2}}+\left[{\text{S}}_{2}\right]\right)\left(1+\frac{\left[\text{P}\right]}{K_{i}}\right)}$$ (4)

We computed the WaaA *k*_*cat*_ value as 16.7 s^-1^ from the specific activity of the purified protein **[14]**. We computed the inhibition constant from results by Belunis and Raetz **[40]** which showed that 100 μM of lipid A inhibited the WaaA reaction by 24.1% *in vitro*. Their experimental conditions involved purified enzyme and substrates, thus excluding the possibility of FtsH playing a role in the inhibition. Whilst assuming a non-competitive inhibition (Eq 4), in which case *K*_*m*_ is constant, we derived that at 100 μM of inhibitor and excess CMP-KDO, a 24.1% reduction in the reaction rate implies that *K*_*i*_ is about 0.0317 mM.

#### LpxC and WaaA synthesis and degradation

We included translation and degradation reactions for LpxC and WaaA in our model so that we could explore the effects of their regulation via FtsH proteolysis. As in the rest of the model, we accounted for degradative protein turnover within cells, but not protein loss through sequestration into daughter cells or the translation that is required to replace those proteins.

We modeled LpxC and WaaA synthesis with zeroth order reaction kinetics, in which the production rate is constant. This approach combines transcription, translation, and any translocation into a single reaction step. We modeled the degradation of these proteins with a first order reaction for degradation that is not catalyzed by FtsH, and also an independent reaction obeying mass action kinetics for degradation that is catalyzed by FtsH (Michaelis-Menten kinetics might be more appropriate, but those parameters cannot be computed from available data). Together, these processes combine to give the net production rate for each of these proteins as

$$\frac{d\left[\text{P}\right]}{dt}=k_{translate}-k_{degrade}\left[\text{P}\right]-k_{FtsH}\left[\text{FtsH}*\right]\left[\text{P}\right]$$ (5)

where [P] is the concentration of the LpxC or WaaA protein, *k*_*translate*_ is the production rate constant, *k*_*degrade*_ is the rate constant for uncatalyzed degradation, and *k*_*FtsH*_ is the rate constant for FtsH degradation.

To determine the production and degradation parameters for LpxC, we started with results presented in Schäkermann *et al* **[14]** which showed that LpxC in wild-type *E*. *coli* can have a half-life of 120 minutes under nutrient and temperature conditions that lead to rapid growth. We assumed that the long LpxC half-life arose because FtsH was essentially inactive under these conditions. This implies that the uncatalyzed LpxC degradation has a half-life of about 120 minutes and *k*_*degrade*_ is about 9.62×10^–5^ s^-1^. Separately, it has been shown that cells with inhibited FtsH activity, using a temperature sensitive mutant, exhibit 4-fold elevated LpxC concentrations **[47]**, thus increasing LpxC counts from about 385 molecules to about 1540 molecules. Combining this molecule count with the *k*_*degrade*_ value and the assumption that [FtsH*_LpxC_] equaled zero in this mutant, enables Eq 5 to be solved for steady-state conditions to give a *k*_*translate*_ value of about 0.148 molec./s. Next, combining the *k*_*degrade*_, *k*_*translate*_, and the wild-type LpxC count of 385 molecules enables Eq 5 to solved for steady-state to give that *k*_*FtsH*_[FtsH*_LpxC_] is 2.89×10^–4^ s^-1^. We cannot solve for *k*_*FtsH*_ by itself from the available information, but estimate its value below.

We computed the WaaA synthesis and degradation parameters similarly. First, a mutant without FtsH maintained about 72% of its WaaA concentration after 30 minutes **[50]**, from which we computed that *k*_*degrade*_ is about 1.8×10^–4^ s^-1^. Next, the half-life of WaaA in wild-type cells under optimal growth conditions is about 10 minutes **[50]**. This gives the combined degradation rate constant, *k*_*degrade*_ + *k*_*FtsH*_[FtsH*_WaaA_], as approximately 1.15×10^–3^ s^-1^, implying that the catalyzed degradation rate constant is about 9.8×10^–4^ s^-1^. Combining the total degradation rate with the wild-type WaaA abundance of 153 molecules per cell gives the protein translation rate, *k*_*translate*_, as 0.176 molecules/s.

#### FtsH activation and inactivation

Our model treats FtsH activation in a substrate-specific manner, but with the constraint that the total FtsH count per cell is conserved at 579 molecules **[62]**. We did not account for FtsH sequestration through activation for other degradation targets, such as RpoH or misfolded proteins. We modeled FtsH activation and inactivation with mass action kinetics, meaning that we treated net FtsH activation towards a specific substrate according to

$$\frac{d\left[\text{FtsH}*\right]}{dt}=k_{active}\left[\text{activator}\right]\left[\text{FtsH}\right]-k_{inactive}\left[\text{FtsH}*\right]$$ (6)

where [FtsH*] represents the concentration of a substrate-specific active form of FtsH, [activator] represents the concentration of the substrate-specific activator (lipid A disaccharide for the LpxC substrate and KDO_2_-lipid A for the WaaA substrate), and [FtsH] represents the concentration of inactive FtsH. The first term on the right hand side represents the activation rate and the second represents the inactivation rate.

The FtsH proteolysis rate, for either LpxC or WaaA, depends on three parameters, *k*_*active*_, *k*_*inactive*_, and *k*_*FtsH*_. However, the available experimental data only enabled us to quantify the product *k*_*FtsH*_[FtsH*], for each substrate, with values given above. Thus, the system is underdetermined, with multiple possible combinations of parameters values that are each equally good at agreeing with the available data. We addressed this by making a few assumptions. First, we assumed that during growth on rich media, 10% of the total FtsH is activated for degradation of LpxC, 10% is activated for degradation of WaaA, and 80% is inactive (i.e. there are 58 copies of FtsH*_LpxC_, 58 copies of FtsH*_WaaA_, and 463 copies of inactive FtsH). This is intuitively sensible because it assumes a reasonably large reservoir of inactive FtsH to allow for strong regulatory control and other proteolysis tasks. Combining this assumption with the prior values for the *k*_*FtsH*_[FtsH*] products yield *k*_*FtsH*_ of 2.0 mM^-1^s^-1^ for LpxC and *k*_*FtsH*_ of 6.8 mM^-1^s^-1^ for WaaA. Secondly, we assumed that both *k*_*inactive*_ values equal 0.1 s^-1^. This gives the active states a 10 s lifetime, which is fast enough to enable rapid control. We then solved for *k*_*active*_ from the steady-state version of Eq 6, while substituting in this inactivation rate constant, the assumed FtsH* and FtsH concentrations, and the activator concentrations that arose from simulations in which we fixed the LpxC and WaaA enzyme counts to the values listed in Table 1 (activator counts were 35,600 and 155 molecules, respectively). Results are that *k*_*active*_ is 0.14 mM^-1^s^-1^ for LpxC and 32.3 mM^-1^s^-1^ for WaaA. Note that the assumptions made here do not affect the model’s steady-state condition at all, making them necessary for running simulations but irrelevant to the results that we present below.

## Results

### Model adjustment

Our initial model, defined using literature parameter values where available and our best estimates elsewhere, exhibited a lipid A production rate that was much too low. Also, several internal metabolite concentrations accumulated to very high levels. We addressed these problems with two model adjustments.

#### LpxM enzyme count

Our initial model simulations only produced about 20% of the 1 million lipid A molecules that *E*. *coli* cells actually produce per generation. This did not change even if we removed FtsH degradation of LpxC and WaaA and all negative feedbacks. We found that this slow production rate arose from LpxM acting as a bottleneck in the pathway, as seen by its substrate increasing linearly over time, rather than stabilizing at a steady-state level. This may indicate that cells have more than the 186 LpxM proteins that proteomic research on the cell cytoplasm indicated **[62]**, which would not be surprising because LpxM is an integral membrane protein. Alternatively, it may be that other enzymes acylate LpxM’s substrate in parallel to LpxM; in particular, LpxL and LpxP can catalyze essentially the same lipid A synthesis reaction **[43]**, **[45]**. Assuming the former explanation, we increased the LpxM molecule count 20 fold, from 186 to 3720. This removed substrate accumulation upstream of LpxM and caused the model to produce lipid A at about 1 million molecules per generation.

#### LpxH product inhibition

Our initial simulations also resulted in high lipid X concentrations, which rose over the course of several cell generations and stabilized at about 400,000 copies. In contrast, 2000 copies were observed *in vivo* **[77]**. We found that the accumulation arose because LpxH rapidly diverted UDP-2,3-bis(β-hydroxymyristoyl)-D-glucosamine towards lipid X, while LpxB only consumed lipid X in a 1:1 ratio with UDP-2,3-bis(β-hydroxymyristoyl)-D-glucosamine. For this reason, we assumed that LpxH is regulated through product inhibition, as described above. The experimental lipid X count was reproduced when we set *K*_*i*_ to 0.2 μM; however, this value is unusually small and it created a bottleneck in the pathway, which we observed as accumulation of the LpxC product and decreased lipid A production (negative feedback at LpxD prevents accumulation of its product, backing the accumulation up to the LpxC product instead). Thus, we decided to decrease LpxH inhibition by increasing *K*_*i*_. As mentioned above, mutants with inactive FtsH exhibit 4-fold increased LpxC concentrations **[47]**; they were also shown to produce 32% more lipid A (Table 1 of **[47]**, comparing their AR3317 at 30°C vs. 42°C, or their AR3289 vs. AR3291). We decided to set the LpxH inhibition constant so that our model would reproduce this result, which turned out to be a *K*_*i*_ value of 0.015 mM. This value was large enough that it did not cause the LpxC product to accumulate in wild-type cells. However, the LpxC product still accumulated in cells without FtsH due to the higher metabolite flux through the LpxC step and the lack of product inhibition at this step. This *K*_*i*_ value caused the steady-state lipid X concentration to be about 22,000 molecules, in either wild-type or FtsH mutant cells, which is much larger than the 2000 that were observed experimentally **[77]**. We found that this difference cannot be eliminated simply by adjusting enzyme kinetic parameters without creating large metabolite accumulations, which suggests that this region of the biosynthesis pathway includes dynamics that are not in our model. For example, the difference could arise from metabolic channeling between the LpxH and LpxB enzymes.

### Comparison of model with experiment

#### Mutant with defective LpxA

Anderson et al. **[27]** showed that cells that have defective LpxA copies, which have at least 150-fold lower LpxA specific activities, have 5- to 10-fold increased LpxC concentrations and an LPS content that is reduced by approximately 30% (their strain SM101 at 30°C). We modeled this perturbation by decreasing the LpxA *k*_*cat*_ value by 150 fold, to 0.048 s^-1^. Our simulation resulted in a 3.9 fold increase in LpxC levels, in reasonably close agreement with experiment. This is essentially the maximum LpxC increase that our model can produce under any condition, arising from our assumption that cells with completely inactive FtsH exhibit 4-fold higher LpxC concentrations **[47]**. However, our model only produced 24,000 lipid A molecules per generation, rather than the 1.1 million molecules that it produces with our standard parameters (Table 1), which is a reduction of 98%. Our results were unaffected by whether we decreased the LpxA reverse reaction *k*_*cat*_ value or not, or even if the reverse reaction was removed altogether. This limitation is clarified by noting that the reaction velocity of the LpxA step is simply *k*_*cat*_[LpxA] in these conditions, from Eq 1, which works out to 57,000 product molecules per generation; these need to dimerize to form lipid A, meaning that the LpxA step limits lipid A production to only 29,000 molecules per generation, in close agreement with our model result. Thus, Anderson et al.’s **[27]** observation that LpxA in the SM101 strain exhibits a 150-fold reduced *k*_*cat*_ value but reduces the lipid A production rate by only 30%, is not compatible with our model. This is because their results cannot arise from our assumed LpxA protein count or *k*_*cat*_ parameters. The disagreement implies that these parameters are incorrect by more than an order of magnitude, which seems unlikely, or potentially, there is an alternate biosynthetic pathway; for example, perhaps LpxD can catalyze what is normally the LpxA step.

#### Inhibition of LpxC

**Fig 4:**
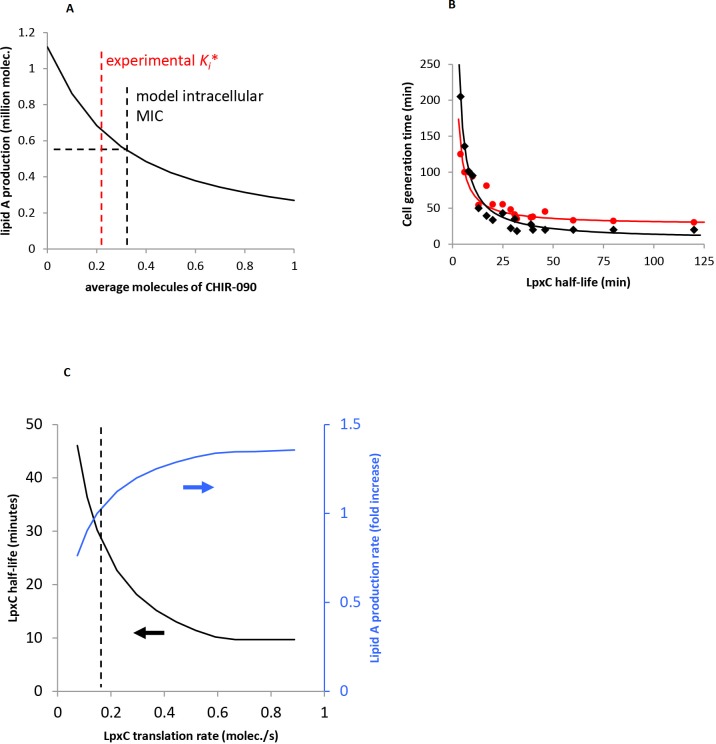
Comparison of model with experiment. (A) Effect of CHIR-090 antibiotic on lipid A production. The model (Fig 2 plus Eq 7) was started with all metabolites at their steady-state concentrations without CHIR-090. Then, antibiotic was added and the total amount of lipid A produced over the following 30 minutes was quantified, shown with the solid black line. The free antibiotic concentration, quantified as the average number of uncomplexed CHIR-090 molecules/cell, was kept constant. The black dashed line shows our estimate of the MIC for the intracellular antibiotic concentration and the red dashed line shows the antibiotic inhibition constant **[9]**. (B) Correlation between LpxC half-life and cell generation time. The experimental data (red circles) are from Schäkermann et al. **[14]**, who varied generation times using different growth conditions and then quantified LpxC half-lives. The model data (black diamonds) were collected by varying the LpxC half-lives (and LpxC *kcat*) and then quantifying the generation times, defined here as the time required to produce 1 million lipid A molecules. Lines are least-difference best-fits to the data using the function *y* = *c*_1_/*x* + *c*_2_, primarily to guide the eye. (C) Effect of overexpressing LpxC on the LpxC half-life (black curve, left axis) and on the lipid A production rate, measured relative to the wild-type production rate (blue curve, right axis). The dashed line shows the wild-type condition using the LpxC translation rate from Table 1.

CHIR-090 is a powerful antibiotic that controls *E*. *coli* and *Pseudomonas aeruginosa* growth with efficacy that is similar to the popular drug ciprofloxacin **[30]**. Its minimum inhibitory concentration (MIC) on *E*. *coli*, meaning the lowest drug concentration required to inhibit visible growth, is between 0.20 and 0.25 μg/ml **[9], [20]**. Barb et al. showed that CHIR-090 acts by inhibiting LpxC through the two-step mechanism **[9]**

$$\text{LpxC}+\text{I}\overset{k_{3}}{\underset{k_{4}}{⇌}}\text{LpxC}-\text{I}\overset{k_{5}}{\underset{k_{6}}{⇌}}\text{LpxC}-\text{I}*$$ (7)

where LpxC-I represents a complex between LpxC and the CHIR-090 inhibitor, and I* represents an enzyme/inhibitor isomer **[9]**. They reported the reaction kinetics parameters as *k*_4_/*k*_3_ = 4 nM, *k*_5_ = 1.9 min^-1^, and *k*_6_ = 0.18 min^-1^ **[9]**. They did not report separate parameters for *k*_3_ and *k*_4_, but their results only becomes logical if this complexation reaction comes to equilibrium reasonably quickly. Thus, we assume here that *k*_4_ = 0.1 s^-1^, from which we compute that *k*_3_ = 25000 mM^-1^s^-1^. We added this mechanism to our model to see if it would exhibit the same inhibitory effect. Firstly, we noticed that fixing the free inhibitor concentration to a constant value does not affect the steady-state biosynthesis pathway at all. This seems reasonable because the LpxC count is not fixed in our model, but arises from the LpxC translation and degradation rates, which the inhibitor does not affect (the reversibility of inhibitor binding implies that, at steady-state, LpxC is sequestered into and released from complexes at the same rate). On the other hand, the inhibitor induces a very strong transient effect. Fig 4A shows the amount of lipid A produced by the model over the course of 30 minutes, in which all metabolite concentrations started at steady-state and then CHIR-090 was added at time 0. We assumed that the CHIR-090 MIC corresponds to the inhibitor concentration that reduces lipid A production by 50%, based on results that show that this is when cells become non-viable **[11]**. From the data shown in Fig 4A, our model cells became non-viable when the free intracellular CHIR-090 concentration exceeds 0.31 molecules/cell, which is 0.76 nM. Clearly, molecule counts are discrete, implying that these are time-averaged quantities. This MIC corresponds closely with the *in vitro* inhibition constant of CHIR-090, *K*_i_*, which is 0.5 nM **[9]**. Taking the *in vivo* MIC as 0.25 μg/ml, and using the CHIR-090 molecular weight of 437.4 g/mol, shows that the MIC is 570 nM of extracellular CHIR-090. Combining this with the intracellular MIC suggests that the intracellular CHIR-090 concentration is about 1000 fold lower than the extracellular concentration.

#### Correlation between LpxC half-life and cell generation time

Schäkermann *et al*. **[14]** showed that faster growing cells, such as those grown in rich media and/or at higher temperatures, stabilize LpxC more rapidly than slowly growing cells. We simulated these experiments as a way of validating our model. We only considered Schäkermann et al.’s results for rich medium because all of our model parameters were estimated from experiments performed with rich medium. For each LpxC half-life value from Table 2 of Schäkermann et al. **[14]**, we used Eq 6 to compute the concentration of FtsH*_LpxC_ that would produce it (here, *k*_*translate*_ was 0, and *k*_*degrade*_ and *k*_*FtsH*_ were the values from Table 1). Next, we changed our standard model in two ways: we adjusted the LpxC *k*_*cat*_ value to account for the given growth temperature according to data shown in Fig 2 of Jackman et al. **[10]**, and we adjusted the rate of FtsH activation for LpxC degradation, *k*_*active*_, until the model exhibited the desired FtsH*_LpxC_ concentration. The rationale for the latter change is that cells might regulate LpxC half-lives by altering the FtsH activation rate. Finally, we computed the cell generation time from the steady-state lipid A production rate in this adjusted model, under the assumption that a generation time is determined by how long a cell requires to produce 1 million lipid A molecules. Fig 4B shows that the model results agree reasonably well with the experimental results, which supports our model. We suspect that our model underestimates cell generation times with long LpxC half-lives because it does not account for other cell processes, which may limit cell division rates at these fast growth rates.

#### Overexpression of LpxC

Führer *et al*. **[48],[49]** cloned the *lpxC* gene into an inducible expression vector and then induced with 0.01% or 0.1% arabinose, which overexpressed the LpxC enzyme. They did not quantify the extent of overexpression, but comparable cells increased protein expression by 100 to 200 fold with 0.01% arabinose induction **[78]**. Führer et al. found that LpxC overexpression increased LPS amounts in cells, by about 1.27 fold and 1.7 fold in cells induced with 0.01% and 0.1% arabinose respectively (our estimates from Fig 4 of **[49]**). They also found that overexpressing LpxC with 0.01% arabinose resulted in a protein half-life of about 11 minutes **[48]**.

We simulated arabinose induction by increasing our model’s LpxC translation rate constant and observing its effect on the lipid A production rate and LpxC half-life (Fig 4C). As in the experiment, overexpressing LpxC led to increased lipid A production and shorter LpxC half-lives. However, these effects stopped changing once LpxC was overexpressed about 4-fold, in contrast to the 100 to 200 fold overexpression that the experiments may have produced. At higher than 4-fold overexpression, the LpxC product accumulated in our model because LpxD became a bottleneck; this prevented further changes to the LpxC lifetime and lipid A production rate. Nevertheless, if one assumes a more modest experimental overexpression, then our results agree well with the 0.01% arabinose induction experiment. In particular, at 2.8 fold overexpression, our model shows a 1.27 fold increase of lipid A production and a 14 minute LpxC lifetime, which is reasonably close to the 11 minutes that Führer et al. observed **[48]**. The 0.1% arabinose induction experiment is harder to match because this resulted in 1.7 fold more LPS, whereas LpxC overexpression could not produce more than 1.36 fold more lipid A in our model due to LpxD acting as a bottleneck. A possible explanation for this difference is that Führer et al. found that LpxC overexpression led to longer cell generation times **[48]**, in addition to the effects mentioned above. Thus, it may be that cells produce lipid A only 36% faster than normal, but they take twice as long to divide, leading to 1.7 fold more LPS in cells. Together, these results show that our model agrees qualitatively with Führer et al.’s LpxC overexpression experiments, and it could agree quantitatively as well, but this cannot be assessed currently with the available experimental data.

#### Substrate limitation

It is well known that organisms grow more slowly when nutrients are limited. For example, Taniguchi et al. **[79]** observed that *E*. *coli* grown on minimal medium had a 150 minute generation time. Replicating this in our model was straightforward. As above, we used the assumption that a cell generation is the time that the model takes to produce 1 million lipid A molecules. Our model reproduces the 150 minute generation time when any one substrate limits the lipid A production rate, with 16,000 UDP-GlcNAc molecules, or 43 β-hydroxymyristoyl-ACP molecules, or 510 CMP-KDO molecules. It also reproduces the 150 minute generation time when all three substrates are partially limiting, with 80,000 UDP-GlcNAc, 210 β-hydroxymyristoyl-ACP, and 2400 CMP-KDO molecules (about 5 times the prior numbers). Thus, substrate limitation does increase generation times in the model, as expected.

However, these results disagree with those of Schäkermann et al. **[14]** in that the low substrate concentrations in the model led to decreased lipid A disaccharide counts, which caused slower LpxC degradation, whereas Schäkermann *et al*. showed that substrate limitation leads to faster LpxC degradation. They found that growth in minimal medium leads to an LpxC half-life of about 10 minutes **[14]**. This appears to occur because substrate limiting conditions lead to increased (p)ppGpp alarmone concentrations, which increase FtsH expression **[80]**. Our model could agree with these data as well, but only if the total FtsH count was increased roughly 25-fold, to about 14,000 molecules (we also used 250,000 UDP-GlcNAc, 650 β-hydroxymyristoyl-ACP, and 7600 CMP-KDO molecules, which are about 15 times higher than the single-substrate limitation values presented above). This modified model exhibited a 10 minute LpxC half-life and a 160 minute generation time, in close agreement with results presented in Schäkermann *et al*. The cost of achieving this agreement was that the high FtsH concentration led to fast WaaA degradation, which made WaaA catalysis of CMP-KDO the rate-limiting step as observed by the fact that lipid IV_A_ accumulated rapidly. The large FtsH expression increase and the lipid IV_A_ accumulation suggest that our model is incorrect for substrate limiting conditions. In particular, achieving the combination of rapid LpxC degradation and relatively low lipid A disaccharide concentrations, without increasing the FtsH concentration 25-fold, requires additional LpxC degradation regulation.

#### Overexpression of LpxK stabilizes LpxC

**Fig 5:**
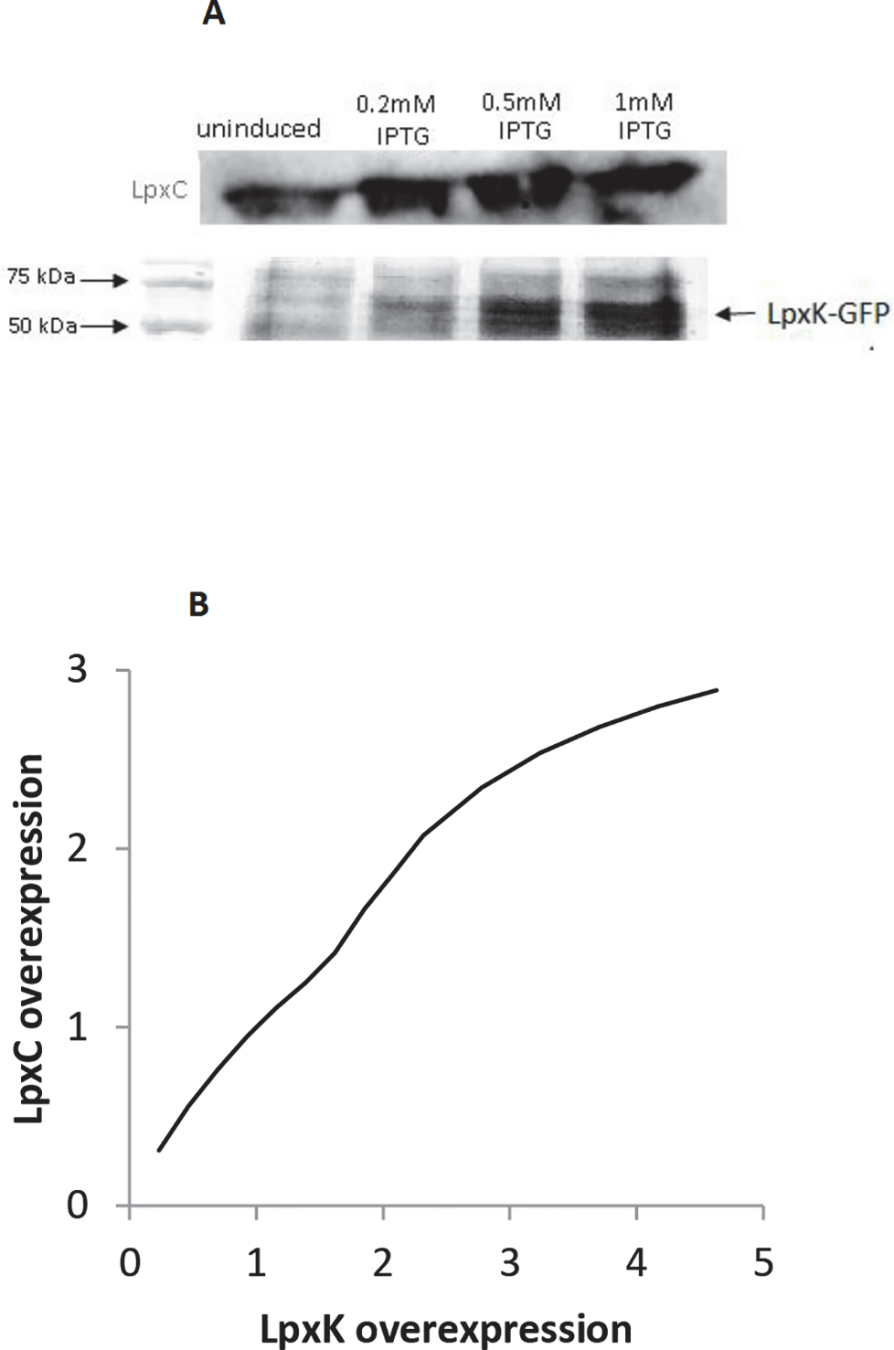
Overexpression of LpxK increases LpxC concentration. (A) Lower row shows LpxK bands on an SDS-PAGE gel, arising from overexpression induced with the amount of IPTG shown at the top of each column. The upper row shows the resultant LpxC bands on a Western blot for the same induction levels. (B) Model prediction of LpxC overexpression arising from LpxK overexpression. The model was that shown in Fig 2 but with different LpxK enzyme counts, at steady-state.

As mentioned above, a preliminary model that did not include FtsH feedback regulation exhibited lipid A disaccharide accumulation (Fig 3), which led us to propose that this metabolite is the feedback source for LpxC degradation. If this is the case, then it follows that LpxK overexpression should reduce lipid A disaccharide concentration, which would down-regulate LpxC degradation and lead to higher LpxC concentrations. We tested this hypothesis experimentally. Fig 5A shows that this is indeed the case. LpxC concentrations increased substantially with LpxK overexpression, even under modest IPTG induction. We tested the same perturbation in our model, finding exactly the same results (Fig 5B). These results are consistent with the assignment of lipid A disaccharide as the feedback source. In contrast, the opposite correlation between LpxC concentration and LpxK overexpression would be expected if the feedback source were downstream of LpxK, so our experiments provide strong evidence against that possibility. Similarly, only a very weak correlation would be expected if the feedback source were further upstream of LpxK, meaning at or before the lipid X metabolite, due to the near irreversibility of the LpxB enzyme, so our experiments provide strong evidence against those possibilities as well. Thus, our experiments strongly indicate that lipid A disaccharide is the feedback source for activating FtsH for LpxC degradation.

### Model predictions

#### Lipid A synthesis sensitivity on enzyme concentration

**Fig 6:**
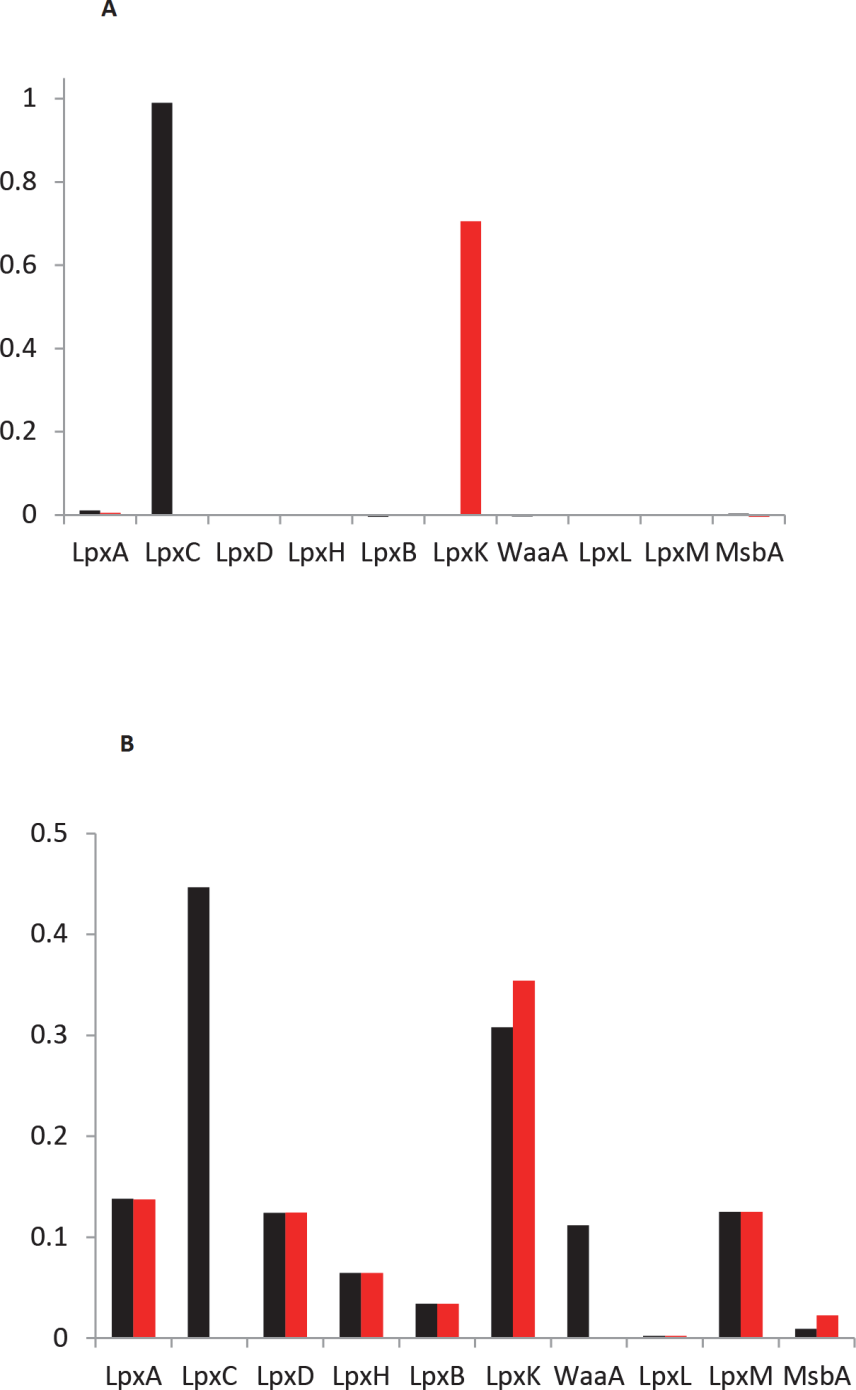
Sensitivity of lipid A production rate on enzyme abundance. (A) Black bars show enzyme control coefficients for the open-loop case, in which wild-type enzyme counts were assumed but FtsH regulation was disabled. Red bars show enzyme control coefficients for the closed-loop case, in which FtsH regulation was enabled. (B) Enzyme abundance reductions that led the model to produce 0.5 million lipid A molecules per cell generation for the open-loop (black bars) and closed-loop (red bars) cases.

We investigated the sensitivity of lipid A synthesis on enzyme counts in several ways. First, we investigated the effect of small enzyme concentration variations. Using the methods of metabolic control analysis **[70], [81]**, we defined flux control coefficients as

$$C_{i}=\frac{\partial \text{ln}\;J}{\partial \text{ln}\;E_{i}}\cdot 100\%$$ (8)

where *i* represents one of the 10 pathway enzymes, *J* represents the biosynthesis rate of lipid A at the outer membrane, and *E*_*i*_ represents the count of enzyme *i*. We quantified flux control coefficients by starting the model at steady-state, varying an enzyme count by 5%, and observing the effect on the lipid A production rate. When we set the three pathway substrates to saturating concentrations and used the “open-loop” case, in which we removed negative feedback through FtsH but kept the wild-type enzyme counts from Table 1, the pathway flux was solely controlled by LpxC (Fig 6A black bars). This is consistent with the view that LpxC is the rate-limiting step of lipid A synthesis **[48],[55]**. However, the “closed-loop” case, in which we replaced the feedbacks through FtsH and thus returned to a better model for the wild-type pathway, showed no sensitivity to LpxC concentration perturbations (Fig 6A red bars). Instead, the feedback caused the LpxC enzyme count to return to its steady-state level, which meant that the perturbation did not affect the lipid A production rate. LpxK was the sole enzyme that controlled pathway flux in the closed-loop case.

Yet different enzymes controlled pathway flux when we reduced substrate concentrations. For each substrate, we set its concentration to the value that led to about 0.5 million lipid A molecules produced per generation and then computed flux control coefficients for each enzyme. Limiting UDP-GlcNAc shifted control to LpxA. This contrasts the view that LpxA does not affect pathway flux, simply because it is reversible and has an unfavorable equilibrium constant **[27]**. Limiting β-hydroxymyristoyl-ACP shifted control to LpxD, LpxH, and LpxB (60%, 20%, and 20%, respectively). Finally, limiting KDO-CMP shifted control to WaaA in the open-loop case, and to MsbA in the closed-loop case. These results show that control of pathway flux is typically localized to relatively few enzymes, but ones which depend on the substrate concentrations.

Next, we investigated the sensitivity of lipid A biosynthesis on enzyme counts for the case of large perturbations, assuming saturating substrate concentrations. For each enzyme, we determined what fraction of the wild-type count (Table 1) would lead to 0.5 million lipid A molecules produced in 30 minutes. In the open-loop case, the production rate was most sensitive to the LpxC and LpxK enzyme counts (Fig 6B black bars). In the closed-loop case, as before, LpxC perturbations were ineffectual because its concentration was regulated through feedback. As a result, the pathway was most sensitive to LpxK (Fig 6B red bars).

#### WaaA Regulation

**Fig 7:**
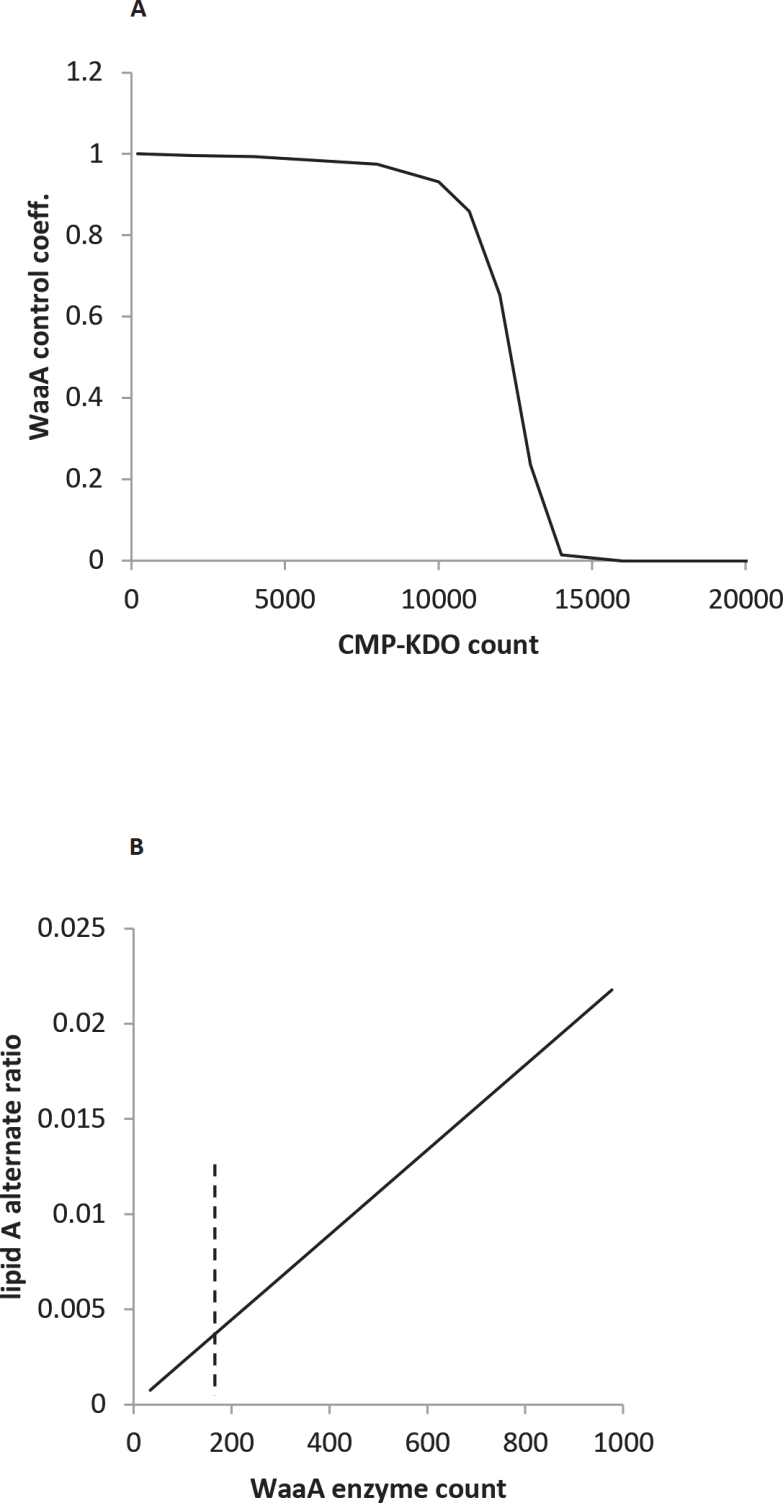
WaaA regulation. (A) The flux control coefficient of WaaA as a function of the CMP-KDO substrate concentration. This is for the open-loop case, in which wild-type enzyme counts were assumed but FtsH regulation was disabled. (B) The ratio of alternate lipid A to normal lipid A (KDO_2_-lipid A) as a function of the number of WaaA proteins. All parameters are the same as in Table 1, except that the WaaA proteolysis rate constant was changed in order to alter the WaaA degradation rate and hence the WaaA steady-state copy number. The maximum enzyme count shown arose from no FtsH mediated proteolysis. The dashed line indicates the wild-type count, from Table 1.

Katz and Ron’s finding that the concentration of WaaA is regulated through degradation by FtsH **[50]** leads to the obvious question of why it is regulated in addition to LpxC. From our sensitivity analysis, one answer may be that WaaA regulation is used to control pathway flux when the CMP-KDO concentration is limiting (Fig 7A). In this situation, the lipid A synthesis rate is insensitive to small changes in the LpxC concentration, making that less useful for regulation, but is controlled by the WaaA concentration instead. Also, reducing lipid A production when CMP-KDO is limiting would conserve KDO for other uses. For example, KDO can be catalyzed by KDO aldolase to produce D-arabinose and pyruvate **[82]**. Although it has been suggested that CMP-KDO synthesis is the rate-limiting step to lipid A synthesis **[83]**, the strong effects of LpxC and the other upstream enzymes, discussed above, indicate that this is not the normal case. Additionally, our model shows that CMP-KDO limitation leads to rapid lipid IV_A_ accumulation, with no apparent correction mechanism. Together, these results point to the likelihood that WaaA regulation helps control the lipid A production rate when the CMP-KDO concentration is low, but probably not so low as to be rate-limiting.

A second possible explanation for WaaA’s regulation is that it might decrease reactions with undesirable substrates **[50]**, brought about by the fact that WaaA has a low substrate specificity. As mentioned previously above, WaaA can glycosylate a wide range of lipid acceptors, including KDO_2_-lipid A in particular **[39], [40]**, which we included in our model architecture as production of alternate lipid A. We ran simulations with different rate constants for WaaA degradation by FtsH. We found that slower WaaA degradation led to higher steady-state enzyme counts, which then led to higher relative amounts of alternate lipid A (Fig 7B). Thus, our model agrees with suggestions that WaaA regulation might help regulate the lipid A molecule composition **[50]**. This mechanism functions even if CMP-KDO is in excess.

## Discussion

We constructed a model of the *E*. *coli* lipid A biosynthesis pathway, including its regulation, using parameters derived from published experimental data. After increasing the LpxM copy number and adding product inhibition to LpxH, this model agreed well with the observed lipid A production rate and exhibited steady-state metabolite concentrations. This model also agreed qualitatively with all of the experiments that we investigated, including ones in which LpxA was defective, LpxC was inhibited with an antibiotic, LpxC lifetimes were compared with cell generation times, LpxC was overexpressed, and substrate concentrations were limited. The model also agreed well with experiments in which we overexpressed LpxK. We are not aware of any published experiments that our model would be expected to disagree with. From this model, we found that the lipid A biosynthesis rate is controlled by LpxC, but only if substrates are in excess and if feedback regulation is ignored. However, LpxK becomes the controlling enzyme if feedback regulation is included, as it is in living cells, and other enzymes gain control if substrate concentrations are below saturation levels. We also found that WaaA may be regulated in order to control the lipid A production rate when the CMP-KDO concentration is limiting, and/or to control the ratio of normal to alternate lipid A that is produced.

Quantitative disagreements between our model and experiments are particularly useful because they show which model assumptions are likely to be incorrect. These errors then enable new insights about the true system behaviour. In particular: (*i*) Our initial model produced lipid A at 20% of the observed rate and exhibited LpxM substrate accumulation, which suggest that LpxM is actually present at a much higher concentration than was measured with proteomic methods **[62]**. (*ii*) High modeled levels of lipid X suggest that the LpxH kinetics are regulated by product inhibition, and also that LpxH and LpxB may form a complex that performs metabolic channeling. (*iii*) The model exhibited 4-fold increased LpxC when LpxA was made defective, instead of the observed 5–10 increase **[27]**; this may arise from experimental differences, or due to unmodeled aspects of LpxC regulation, such as transcription regulation. (*iv*) The model exhibited a 98% reduction in the lipid A synthesis rate when LpxA was defective, instead of the 30% that was observed experimentally **[27]**, which suggests the presence of alternate metabolic routes around LpxA, such as by LpxD. (*v*) The model underestimated cell generation times when LpxC had a very long half-live **[14]**, which suggest that the growth rates of fast growing cells are not limited by the lipid A production rate. (*vi*) The model could exhibit the experimentally observed LpxC half-life and cell generation time when substrates were limited **[14]**, but only when the total FtsH concentration was increased 25-fold; this suggests that there are additional mechanisms that regulate LpxC degradation. None of these speculations are proven by the model. Instead, they are possible solutions to situations in which the experiments that we drew on to create the model do not agree with experiments that were used to test the model. They provide hypotheses for further experimental investigation.

The combination of prior experimental results, our model, and our LpxK overexpression experiments provide strong evidence for lipid A disaccharide being a primary feedback source for LpxC degradation (Fig 5A). Furthermore, these same sources of information suggest that this is the only feedback source among the chemical species that we modeled. However, there is also evidence for other LpxC regulation mechanisms. As mentioned previously above, the LpxC concentration also appears to be regulated by signals arising in the phospholipid synthetic pathway **[20],[24],[47]**. Also, the substrate limitation studies mentioned above suggest the presence of additional regulation mechanisms. Together, these point to substantial signal processing taking place at FtsH, which then controls lipid A synthesis through LpxC.

Yet more pathway regulation may take place in other ways. For example, Ray and Raetz **[35]** found that phospholipids, and especially cardiolipin, increase the catalytic activity of LpxK. Based on our experiments, this increase would lead to higher LpxC concentrations and hence faster lipid A production, which could help balance the phospholipid to LPS ratio. As another example, overall protein production is slowed during substrate limiting conditions **[84]**, which undoubtedly reduces the concentrations of the lipid A synthesis enzymes and hence reduces the lipid A synthesis rate. The significance of this effect was illustrated by Zeng et al. **[20]**, who found that strains that possess mutations in the *thrS* gene, whose function is vital to overall protein synthesis, were resistant to LpxC inhibition.

There are several possible purposes for WaaA regulation. First, it may serve to regulate lipid A production, which our model showed is possible, but only if CMP-KDO is at least partially limiting. Also, it may serve to regulate the relative production of lipid A and alternate lipid A **[50]**, which our model also showed is possible. In support of the latter role, some of the precursors for other substrates that can be glycosylated by WaaA could not be detected in wild-type *E*. *coli* **[77]**, suggesting that this step is tightly controlled. The situation changes at elevated temperatures, where these alternate substrates accumulate **[85]** and, probably not coincidentally, WaaA is degraded faster under these conditions **[50]**. Furthermore, it is well established that the composition of lipids within bacterial membranes are altered as a response to temperature fluctuations and growth rate changes **[86], [87], [88]**, presumably to control membrane fluidity. Yet another proposed purpose for WaaA regulation is to balance the synthesis of the sugar and lipid moieties of KDO_2_-lipid A **[50]**. However, this explanation seems unlikely because the uptake of these moieties by WaaA is always 2:1, independent of its regulation.

Our model can help elucidate which lipid A pathway enzymes are likely to be good or poor antibacterial targets. Most of the best known inhibitors of the LPS pathway to date have been directed at LpxC **[11], [30], [89]**, including the CHIR-090 antibiotic discussed above, presumably because most intracellular synthesis regulation takes place at LpxC. However, our sensitivity analysis showed that this intracellular regulation may actually make LpxC a poor drug target because it enables the cell to counteract external perturbations. Indeed, it was recently reported by Walsh and Wencewicz **[90]** that the development of CHIR-090 has been hampered due to the ease of pathogen mutation to resistance. On the other hand, our model suggests that LpxK would make a good target, as initially suggested by Emptage *et al*. **[91]**. LpxK is an essential enzyme without alternative synthetic routes, so strong inhibition would arrest lipid A production. Also, LpxK does not appear to be regulated (except possibly by cardiolipin **[35]**), which might make cells unable to counteract its inhibition. Additionally, when we accounted for feedback regulation, we found that the lipid A production rate is particularly sensitive to the LpxK concentration (Fig 6). And finally, inhibiting LpxK would lead to lipid A disaccharide accumulation. Lipid A disaccharide is cytotoxic **[65]** and its accumulation would lead to LpxC down-regulation, which would further repress lipid A production.

A central theme of cell biology modeling method development is that non-spatial, non-stochastic models are too simplistic **[92],[93]**. In response, new software tools offer support for stochastic reaction dynamics and spatial localization of proteins. In this case, we started this research using the Smoldyn simulator **[16]**, which accurately addresses stochasticity and spatial detail, but found that simulations ran too slowly and the spatial detail did not affect the results. Thus, we switched to StochKit **[17]**, which performs stochastic simulations but ignores spatial localization. Again, the stochastic detail proved unnecessary. We finally settled on non-spatial deterministic simulations. These simple simulations were appropriate in this case because the lipid A pathway is sufficiently poorly parameterized through experimental work. Therefore, including additional detail would have only complicated the model further. However, more detailed simulation methods will likely become useful as the lipid A synthesis becomes better characterized. For example, we showed that time-averaged intracellular CHIR-090 antibiotic count in cells was about 0.3 molecules, implying that it is present in extremely low copy numbers. This could lead to strong stochastic effects. Also, our results suggest the presence of interesting dynamics between LpxH and LpxB, such as metabolic channeling. These might be best modeled using spatial simulation methods.

In conclusion, we present a quantitative model of lipid A biosynthesis and its regulation. The core biosynthesis pathway agrees with the commonly accepted architecture, while the regulation elements are largely new. Our model agrees qualitatively with a wide range of experimental results, but also shows substantial quantitative differences. Thus, our model is not a final picture of lipid A biosynthesis, but instead represents the best understanding of lipid A synthesis available to date.

## Supporting Information

S1 File COPASI file of lipid A biosynthetic model in *E*. *coli* using parameters described in Table 1. (CPS)

S2 File COPASI file modelling the effect of CHIR-090 antibiotic on lipid A production. Parameters are same as in S1 with the inclusion of Eq 7.
(CPS)
